# Linguistic camouflage in girls with autism spectrum disorder

**DOI:** 10.1186/s13229-017-0164-6

**Published:** 2017-09-30

**Authors:** Julia Parish-Morris, Mark Y. Liberman, Christopher Cieri, John D. Herrington, Benjamin E. Yerys, Leila Bateman, Joseph Donaher, Emily Ferguson, Juhi Pandey, Robert T. Schultz

**Affiliations:** 1 Center for Autism Research, Children’s Hospital of Philadelphia, Roberts Center for Pediatric Research, 2716 South Street, Philadelphia, PA 19146 USA; 2 Perelman School of Medicine, University of Pennsylvania, 3400 Civic Center Blvd, Philadelphia, PA 19104 USA; 3 Linguistic Data Consortium, University of Pennsylvania, 3600 Market St #810, Philadelphia, PA 19104 USA; 4 Center for Childhood Communication, Buerger Center for Advanced Pediatric Care, Children’s Hospital of Philadelphia, 3500 Civic Center Blvd, Philadelphia, PA 19104 USA

**Keywords:** Autism, Language, Linguistic camouflage, Disfluency, Pragmatic communication, Sex differences, Gender differences, Filled pauses

## Abstract

**Background:**

Autism spectrum disorder (ASD) is diagnosed more frequently in boys than girls, even when girls are equally symptomatic. Cutting-edge behavioral imaging has detected “camouflaging” in girls with ASD, wherein social behaviors appear superficially typical, complicating diagnosis. The present study explores a new kind of camouflage based on language differences. Pauses during conversation can be filled with words like UM or UH, but research suggests that these two words are pragmatically distinct (e.g., UM is used to signal longer pauses, and may correlate with greater social communicative sophistication than UH). Large-scale research suggests that women and younger people produce higher rates of UM during conversational pauses than do men and older people, who produce relatively more UH. Although it has been argued that children and adolescents with ASD use UM less often than typical peers, prior research has not included sufficient numbers of girls to examine whether sex explains this effect. Here, we explore UM vs. UH in school-aged boys and girls with ASD, and ask whether filled pauses relate to dimensional measures of autism symptom severity.

**Methods:**

Sixty-five verbal school-aged participants with ASD (49 boys, 16 girls, IQ estimates in the average range) participated, along with a small comparison group of typically developing children (8 boys, 9 girls). Speech samples from the Autism Diagnostic Observation Schedule were orthographically transcribed and time-aligned, with filled pauses marked. Parents completed the Social Communication Questionnaire and the Vineland Adaptive Behavior Scales.

**Results:**

Girls used UH less often than boys across both diagnostic groups. UH suppression resulted in higher UM ratios for girls than boys, and overall filled pause rates were higher for typical children than for children with ASD. Higher UM ratios correlated with better socialization in boys with ASD, but this effect was driven by increased use of UH by boys with greater symptoms.

**Conclusions:**

Pragmatic language markers distinguish girls and boys with ASD, mirroring sex differences in the general population. One implication of this finding is that typical-sounding disfluency patterns (i.e., reduced relative UH production leading to higher UM ratios) may normalize the way girls with ASD sound relative to other children, serving as “linguistic camouflage” for a naïve listener and distinguishing them from boys with ASD. This first-of-its-kind study highlights the importance of continued commitment to understanding how sex and gender change the way that ASD manifests, and illustrates the potential of natural language to contribute to objective “behavioral imaging” diagnostics for ASD.

N.B.: In this paper, our terminology is drawn from World Health Organization definitions [51]. The word “sex” refers to genetic makeup, and “gender” refers to a socio-cultural construct. Consistent with a recent review of sex differences in ASD [31], we explicitly acknowledge the significant and inevitable overlap between the two. Here, we use the words “girl” and “boy” to refer to biological sex.

## Background

Autism spectrum disorder (ASD) is a behaviorally defined condition predominantly found in males [10, 19, 40, 49]. Recent research suggests that girls with ASD may “camouflage” real struggles with social communication by engaging in social mimicry and behaving in ways that are superficially typical, thus complicating diagnosis [7, 34]. For example, nonverbal communication (e.g., gesture) is broadly impaired in ASD [3]. Using 3D motion capture, a recent study showed that girls with ASD gesture in ways that are more vibrant and noticeable than boys with ASD, despite similar struggles with social communication [48]. In this way, girls effectively modified their behavior to mask a traditional area of weakness. In the present study, we use granular language-based analysis to explore another way in which girls with ASD achieve this social camouflage effect: by producing sex-typical filled pauses during naturalistic conversation.

### Filled pauses

In the course of normal conversation, interlocutors pause, revise, and re-work their utterances, a process termed *disfluency* [11]. Disfluencies affect how speakers are perceived by others, and elevated rates of speech disfluencies have been linked to negative social perceptions of the speaker [8, 22, 45]. Pauses are disfluencies that occur during natural speech and can either remain unfilled (silent pauses) or be filled by words like *um*, *uh*, *like*, and *you know* (filled pauses)*.* UM and UH, in particular, are thought to be social pragmatic hesitation markers with communicative value (e.g., facilitating comprehension by signaling to the listener that the speaker needs more time to finish communicating their current thought, or that they would like to hold/cede the floor [21]). However, there is growing evidence to suggest that UM and UH are not the same; UH is used to signal a short delay while UM may be used to signal more significant delays [11]. Large-scale cross-linguistic studies of spoken and written language show that these two markers are used with varying frequency by people from different demographic groups [1, 59]. For example, UM is used relatively more often by women, educated individuals, and younger generations than by men, less educated individuals, and older generations. In contrast, UH is used relatively more frequently by men, less educated individuals, and older generations than by women, more educated individuals, and younger generations (M. [37, 59]). This pattern is robust across American English, British English, Scottish English, Dutch, Norwegian, German, Danish, and Faroese [59].

### Filled pauses in ASD

A significant body of research suggests that children and adults with ASD produce unusually high rates of various speech disfluencies [16, 35, 42, 50, 51, 54], but only three studies have specifically examined UM and UH. First, 4- to 8-year-olds with ASD use UM at lower rates than children without ASD [26]. This finding suggests that UM and UH may hinge on distinct cognitive processes, with UM more powerfully affected by the presence of autism [26]. Subsequent research showed that UM use by 8- to 21-year-olds normalizes when overall ASD symptoms drop off [28, 46], again lending support to the hypothesis that UM is associated with social competence. In a third study, language samples from children with ASD were compared to samples from children with specific language impairment, and individual variation in UM use was found to be a marker of pragmatic ability rather than a consequence of general language skill [24]. Taken together, these studies suggest that UM and UH are used differently by children with ASD, and that UM ratios [um/(um + uh)] associate with ASD symptoms.

One major limitation of this line of research is that sex differences in filled pause use have never been studied in individuals with ASD. If females have higher UM ratios than males [59], slight sex ratio differences across diagnostic groups could lead to erroneous results. For example, Gorman et al. [24] included 10% girls in their ASD group and 30% girls in their typical group. Elevated UM ratios in the typical group relative to the ASD group might thus be a consequence of sex ratio imbalances (because girls may produce higher UM ratios than boys)—and the TD group was enriched for girls. This observation, that slight differences in sex ratio by diagnostic group could change the results of scientific studies in a variety of domains, has implications for how we evaluate the presence and severity of ASD symptoms in girls and boys, and will influence the hunt for behavioral markers of ASD more broadly. In the case of UM and UH specifically, if girls with ASD exhibit gender-normative speech patterns, it could bias perception of their ASD symptoms. The current literature is unable to address this question, primarily out of an absence of speech data from females with ASD. Apart from Gorman et al. [24], the other two papers that explored UM vs. UH in children with ASD either did not report sample sex ratios at all [26], or included insufficient numbers of female participants with ASD to assess potential effects of sex on filler production (3 out of 24 or 12.5% in [28]).

The present study fills this gap with a large sample of children on the spectrum, including a representative proportion of girls (*N* = 65, 16 girls, 25%), along with a small comparison sample of typical children (*N* = 17, 9 girls, 53%). We hypothesized that girls and boys with ASD would show typical sex differences in filled pauses (i.e., girls would produce higher UM ratios than boys with ASD, driven by boys producing more UH than girls). We further hypothesized, based on prior research showing that scores on the Social Communication Questionnaire (SCQ; [46] higher scores indicate more lifetime ASD symptoms) decrease as UM ratio increases [24, 28], that higher UM ratios would be associated with fewer lifetime ASD symptoms. To address the possibility that girls in our ASD sample were more socially adept than boys, we compared parent reports of social communication ability by sex (using the Vineland Adaptive Behavior Scales-2nd edition, (VABS; [52]), and explored whether better social communication in girls explained sex differences in filled pause ratios. Finally, we analyzed subgroups of boys with ASD, and gauged the specificity of our UM/UH results by comparing boys and girls on seven additional linguistic features. Language samples were drawn from the interview section of the ADOS module 3 (requiring phrase speech; [41]). These samples provide a naturalistic back-and-forth conversation about social topics, and have been shown to contain meaningful rates of speech disfluencies in children with ASD [24, 26, 42].

## Methods

### Participants

Sixty-five children with ASD aged 6–17 contributed language samples during research visits to the Center for Autism Research at the Children’s Hospital of Philadelphia (Table 1). Children were recruited from the larger Philadelphia area, and 85% of the sample was White according to parent report. Research-reliable clinical psychologists (100% female) used expert clinical judgment to make diagnoses of ASD according to DSM-IV-TR criteria [2] informed by the ADOS-2 Module 3 and the Autism Diagnostic Interview-Revised (ADI-R; [47]). Exclusion criteria included extreme prematurity (< 32 weeks) or low birth weight, genetic or medical history that explained ASD symptoms, uncorrected auditory or visual impairment, significant psychiatric conditions (e.g., active mood disorders or psychosis, but not common comorbidities like anxiety or ADHD), and contraindications for an MRI scan (e.g., braces). Inclusion criteria for the present subsample included the ability to use phrase speech, since all conversational data were drawn from Module 3 ADOS evaluations. Boys and girls did not differ significantly on age, General Conceptual Ability (GCA) using the Differential Abilities Scales-II (DAS-II; all participants had GCAs > 75), or ASD symptoms as measured by ADOS scores (Table 1). Despite statistically insignificant differences in chronological age, there were mean differences; we therefore included this variable in our analysis, to check for possible age influences on filled pauses. In addition, we recruited a small sample of typically developing children (*N* = 17; age in years: *M* = 11.32, SD = 2.21; GCA: *M* = 104, SD = 15) for the purposes of comparison.

**Table 1:** Demographics and clinical scores (mean (standard deviation)) for children with ASD

	Overall	Boys	Girls	Sex difference
Number	65	49	16	33 more boys
Age in years	9.96 (2.05)	9.73 (2.16)	10.66 (1.55)	*Z* = 1.60, *p* = 0.10
DAS-II GCA	105 (14)	106 (14)	104 (13)	*Z* = −0.07, *p* = 0.95
Verbal	107 (14)	107 (14)	106 (15)	*Z* = −0.35, *p* = 0.73
Nonverbal	106 (14)	106 (14)	105 (14)	*Z* = −0.24, *p* = 0.81
SCQ lifetime	19.82 (6.92)	19.49 (7.46)	20.81 (4.98)	*Z* = −1.01, *p* = 0.31
VABS composite	82.32 (13.22)	83.19 (13.26)	79.75 (13.18)	*Z* = −0.85, *p* = 0.39
Communication	87.75 (13.77)	88.21 (14.14)	86.38 (12.94)	*Z* = −0.31, *p* = 0.76
Socialization	77.95 (14.88)	79.36 (15.48)	73.81 (12.49)	*Z* = −1.74, *p* = 0.08
ADOS-2 overall^a^	6.49 (2.47)	6.55 (2.38)	6.31 (2.80)	*Z* = −0.18, *p* = 0.85
Social affect	6.29 (2.42)	6.29 (2.38)	6.31 (2.63)	*Z* = −0.008, *p* = 0.99
RRB	7.08 (2.54)	7.27 (2.32)	6.50 (3.14)	*Z* = −0.43, *p* = 0.67

Sex differences were assessed via nonparametric Mann-Whitney *U* tests. ADOS scores represent calibrated severity scores (comparison scores). Two male participants are missing Vineland Adaptive Behavior Scales (VABS) scores. *RRB* = repetitive behaviors and restricted interests

^a^Calibrated severity scores of 4 are equivalent to a raw score of 7 (ASD cut-off) and severity scores of 6 are equivalent to a raw score of at least 9 (autism cut-off) on the ADOS-2

### Procedure

Research-reliable clinicians administered the ADOS to participants as part of 1- or 2-day study battery (rescored as the ADOS-2, referred to as the ADOS-2 throughout this paper). The ADOS-2 was administered in a quiet room equipped with audio and video recording equipment. Parents consented to using these recordings for research purposes. Participant IQ was estimated using the DAS-II [18], and parents provided additional information via the ADI-R [47], the VABS [52], and the Social Communication Questionnaire (SCQ; [46] see the “Measures” section below). Families were compensated for time and travel. This research was conducted with approval and oversight from the Institutional Review Board of the Children’s Hospital of Philadelphia.

### Measures

All measures were administered in person, by telephone (for the ADI-R, if needed), or via US postal service (for parent questionnaires).

Autism Diagnostic Observation Schedule-2nd Edition (ADOS-2; [41]). The ADOS-2 is a semi-structured psychodiagnostic assessment for ASD, often used in combination with parent history (collected via ADI-R or SCQ combined with parent interview). ADOS-2 calibrated severity scores estimate the extent to which an individual is affected by ASD symptoms (0–10, with 0 representing least affected and 10 most affected [25]). Calibrated severity scores for the subdomains of social affect (0–10) and repetitive behaviors/restricted interests (0–10) were calculated as well [25, 27].

Differential Abilities Scales-II (DAS-II; [18]). The DAS-II is designed to estimate general intelligence in individuals aged 2–18 years. The DAS-II provides standard scores that are analogous with full-scale IQ (DAS-II General Conceptual Ability), verbal IQ (DAS-II verbal composite score), and performance IQ (DAS-II nonverbal composite score).

Social Communication Questionnaire (SCQ; [46]). The SCQ is a parent report questionnaire assessing current and lifetime autism symptoms. This study reports lifetime SCQ scores, with higher scores indicating greater lifetime autism symptomology.

Vineland Adaptive Behavior Scales (parent/caregiver rating form)-2nd edition (VABS; [52]). The VABS is a parent report measure of their child’s adaptive behavior. It includes multiple domains of adaptive function, including socialization and communication. Higher scores indicate more proficient adaptive behavior.

### Transcription

The largest continuous segment of the “interview questions” section of the ADOS-2 (in minutes) was orthographically transcribed using a specification developed in collaboration with the University of Pennsylvania’s Linguistic Data Consortium (LDC). When possible, we transcribed the “Emotions,” “Social Difficulties and Annoyance,” “Friends, Relationships, and Marriage,” and “Loneliness” sections. The “Break” section, which occurs within these sections, was not transcribed. Mean overall transcription length was 19.61 min per participant. Samples from boys (mean = 19.15 min) vs. girls (mean = 20.65) did not significantly differ in duration, Mann-Whitney *Z* = −1.48 *p* = 0.14. Each audio sample was segmented into utterances (separated by breath pauses) by an undergraduate student and checked by an experienced annotator. Speech segments were transcribed by a different undergraduate student, and adjudicated by a different experienced annotator. Segmentation, transcription, and adjudication were conducted using audio files only, blind to diagnosis. Nearly 50% of recordings were fully transcribed by two independent annotators, with pre-adjudication word-level agreement averaging 92%. Time-stamped textual transcriptions were aligned with audio files using a force-aligner developed at the LDC [60].

### Variables

Word count was calculated by summing all words produced by each speaker (totwords). Instances of UM and UH were identified within each transcript, and summed. Three variables were calculated: average UM production (tot_um/totwords), average UH production (tot_uh/totwords), and um_ratio, which is the rate of UM produced relative to total filled pauses [tot_um/(tot_um + tot_uh)]. Total filled pause rate was calculated by dividing the sum of pauses filled by UM or UH by the total number of words produced by an individual [(tot_um + tot_uh)/totwords]. Average latency to respond to the ADOS-2 administrator was calculated by dividing the sum duration of all clinician-to-participant inter-turn pauses by the total number of pauses of that transition type. Speech rate was calculated by dividing the total number of words produced by each speaker by the summed duration of all segments for that speaker. An outlier-robust measure of dispersion in participants’ fundamental frequency (F0) distribution (median absolute deviation from the median), captured pitch variation. All time variables are reported in milliseconds.

### Statistical plan

Three generalized linear mixed effects logistic regression models assessed the relative effects of age, IQ, sex, and ASD symptoms on “tot_um,” “tot_uh,” and “um_ratio” in participants with ASD (software: R, package: lme4; [59]). In each case, the word of interest was coded as a “hit” while all other words were coded as “misses.” Continuous variables were z-scored for interpretability, and participant identity was included with a random intercept. UM and UH variables were non-normal and contained outliers; analyses with and without outliers were found to yield the same pattern of results, so outliers were retained to capture the heterogeneity inherent in samples with ASD. Nonparametric Mann-Whitney *U* tests were used in place of standard *t* tests to account for non-normality (software: SPSS 23; Z is reported). Means and standard deviations are reported for the typically developing comparison group, but due to the small sample size, only nonparametric Mann-Whitney *U* tests were conducted. Spearman’s Rho (*ρ*) was used to characterize correlational relationships between UM and UH variables and questionnaire scores for participants with ASD. Effect sizes for Mann-Whitney *U* tests are reported using *r* = Z/[sqrt(*N*)] [20]. Following Cohen (1988), an *r* value of 0.1 is considered a small effect, 0.3 is a medium effect, and 0.5 is a large effect.

## Results

### Predictors of UM and UH in ASD

Generalized linear mixed effects logistic regression models revealed significant associations between sex, VABS socialization scores and average UH production in the ASD group, as well as UM ratio, but no association between sex and socialization and average UM production (Table 2). Age, GCA, SCQ, and VABS communication scores did not account for significant explanatory variance in the three filled pause variables, nor did interactions between any two variables. The pattern of results did not change when verbal and nonverbal composite scores were entered instead of DAS-II GCA.

**Table 2:** Generalized linear mixed effects regressions predicting average UM and UH relative to total words produced, and UM ratio (UM/(UM + UH))

	Average UM			Average UH			UM ratio		
	Est. (se)	*z*	*p*	Est. (se)	*z*	*p*	Est. (se)	*z*	*p*
Intercept	−4.33 (0.27)	−16.25	< 0.000	−5.98 (.23)	−26.51	< 0.000	−0.14 (.13)	−1.09	0.28
Age	−0.21 (0.16)	−1.38	0.17	−0.15 (0.12)	−1.21	0.23	0.05 (0.08)	−0.58	0.56
GCA	0.08 (0.16)	0.55	0.58	−0.11 (0.12)	−0.95	0.34	−0.04 (0.07)	0.53	0.59
Sex	−0.50 (0.30)	−1.68	0.09	0.82 (0.24)	3.36	< 0.001**	−0.46 (0.15)	−3.18	0.001**
SCQ	0.08 (0.21)	0.37	0.71	−0.02 (0.16)	−0.10	0.92	0.06 (0.11)	0.55	0.58
V Com	−0.12 (0.23)	−0.53	0.60	0.26 (0.17)	1.47	0.14	−0.08 (0.12)	−0.69	0.48
V Soc	0.16 (0.26)	0.62	0.54	−0.69 (0.20)	−3.48	< 0.001**	0.29 (0.13)	2.28	0.02*

Predictors associated with greater dependent variable values have positive log-odds (shown as estimate (Est.) with standard error (se) in parentheses). *V Com* Vineland Communication Domain scores, *V Soc* Vineland Socialization Domain scores, *Sex* 0 = girl, 1 = boy. * *p*<.05, ** *p*<.01

Boys with ASD produced more UH relative to total words produced (mean = 0.0084, SD = 0.0077, 95% CI = 0.0062–0.0106) than girls with ASD (mean = 0.0044, SD = 0.0044, 95% CI = 0.0022–0.0066), mean difference = −0.004, 95% CI = −0.0081–0.00004, *Z* = −1.98, *p* = 0.02, *r* = 0.29. There was also a significant sex difference in UM ratio (mean difference = 19%, 95% CI = 0.04–0.34, *Z* = −2.16, *p* = 0.03, *r* = 0.26). Whereas girls produced UM during approximately 75% of pauses filled by UM or UH (SD = 17%; CI = 0.66–0.85), boys produced UM during an average of 56% of such pauses (SD = 29%, CI = 0.48–0.65). Average UM use did not differ in boys (mean = 0.0143, SD = 0.0156, 95% CI = 0.0098–0.0188) as compared to girls (mean = 0.0161, SD = 0.0125, 95% CI = 0.0094–0.0227), mean difference = 0.0017, 95% CI = −0.0069–0.0103, *Z* = −1.19, *p* = 0.24, *r* = 0.15, suggesting that greater UH use by boys (or reduced UH use by girls) drove the significant difference in the um/uh ratio.

### UM and UH in typical controls

**Fig. 1:**
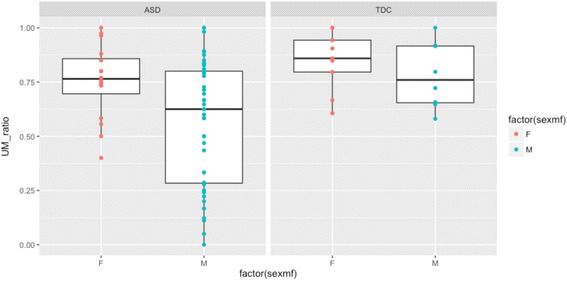
UM ratio for girls (F) and boys (M) with and without ASD. *TDC* ﻿= typically developing control

A similar pattern emerged in our sample of typically developing children. Typical boys produced more UH relative to total words produced (mean = 0.0083, SD = 0.0050, 95% CI = 0.0042–0.0124) than did typical girls (mean = 0.0032, SD = 0.0027, 95% CI = 0.0011–0.0053; mean difference = −0.0051, 95% CI = −0.0092–(−0.0010), *Z* = −2.32, *p* = 0.02, *r* = 0.56; Fig. 1). Similar to the ASD group, mean UM production relative to total words produced was comparable between typical boys (mean = 0.0301, SD = 0.0129, 95% CI = 0.0193–0.0408) and girls (mean = 0.0234, SD = 0.0184, 95% CI = 0.0011–0.0053; mean difference = −0.0067, 95% CI = −0.0233–0.0100, *Z* = −1.06, *p* = 0.29, *r* = 0.26). However, in contrast to the pattern seen in children with ASD, UM ratios were high for both typical girls (mean = 85%, SD = 14%, 95% CI = 74–95%) and boys (mean = 78%, SD = 15%, 95% CI = 65–91%; mean difference = 0.0673, 95% CI = −0.0827–0.2173, *Z* = −0.87, *p* = 0.39, *r* = 0.21.

### Diagnostic group differences in UM and UH

Given our small TDC sample, we suggest that the following results be interpreted with caution. Overall, typical children filled more pauses than children with ASD (Table 3; *Z* = −2.33, *p* = 0.02, *r* = 0.26). Mann-Whitney *U* tests suggest that girls with ASD and typical girls used comparable levels of UM (*p* = 0.34) and UH (*p =* 0*.*61), and had similar UM ratios (*p* = 0.16; Fig. 1). In contrast, boys with ASD produced significantly less UM (relative to total words produced) than typical boys (*p* = 0.002), and typical boys produced higher UM ratios than boys with ASD (*p* = 0.06). Boys in the ASD and TDC groups did not differ on average UH (*p* = 0.63).

**Table 3:** UM use, UH use, and total filled pause rates, by sex and diagnostic group

	Average UM		Average UH		UM ratio		Filled pause rate	
	Mean (%)	SD (%)	Mean (%)	SD (%)	Mean (%)	SD (%)	Mean (%)	SD (%)
ASD								
Girls	1.6	1.2	0.44	0.44	75	17	2.0	1.5
Boys	1.4	1.6	0.84	0.77	56	29	2.3	1.7
TDC								
Girls	2.3	1.8	0.32	0.27	85	14	2.7	1.9
Boys	3.0	1.3	0.83	0.50	78	15	3.8	1.3

### Filled pauses and ASD symptoms

**Fig. 2:**
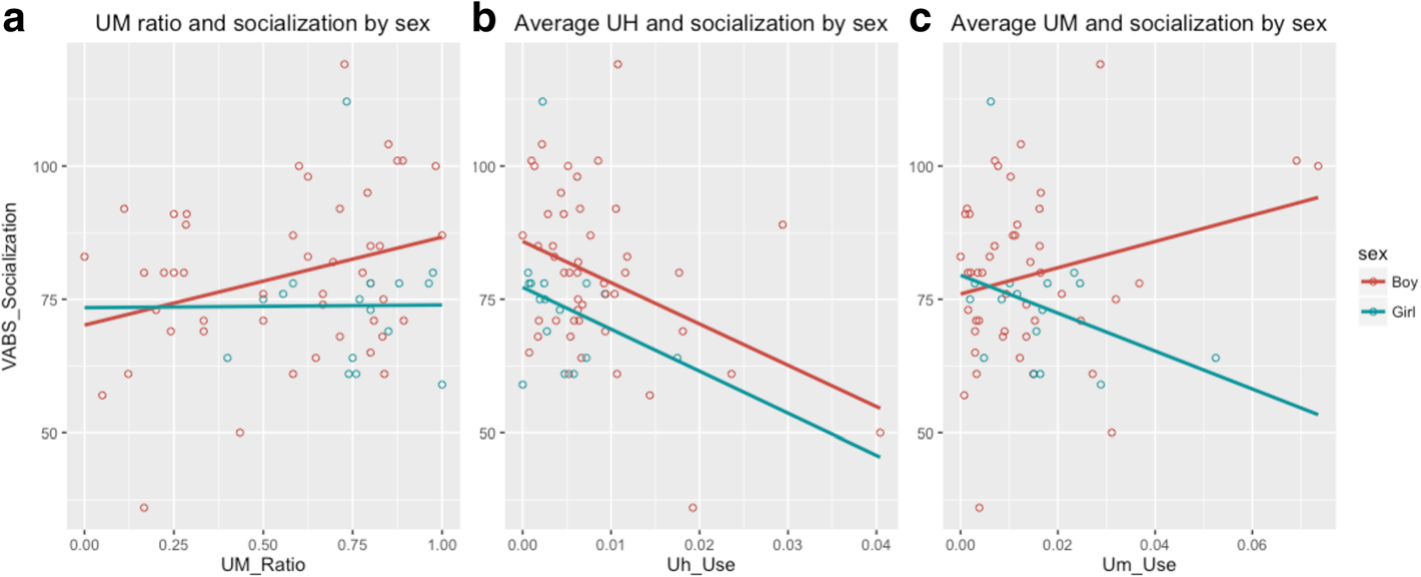
Parent reported VABS socialization scores and UM ratio (**a**), average UH production relative to total words produced (**b**), and average UM production relative to total words produced (**c**) by boys and girls with ASD (female =* blue*; male = *red*). Correlations in **a** and **b** are significant for boys but not for girls; correlations in **c** are not significant, but are included to demonstrate opposite relationships between socialization and average UM production by sex

Generalized linear mixed effects logistic regression models revealed that UM ratio and average UH production are associated with VABS socialization scores in the ASD group. To rule out the possibility that slightly lower VABS scores in girls relative to boys in our sample unduly influenced the observed relationship between socialization and disfluency type, we explored whether relationships between filled pauses and VABS socialization scores held within boys and girls with ASD, separately (Fig. 2). Spearman’s Rho correlations revealed a positive association between VABS socialization scores and UM ratio for boys (*ρ* = 0.30, *p* = 0.04) but not for girls (*ρ* = 0.007, *p* = 0.98; 2a). Increased UH use was associated with lower socialization scores for boys (*ρ* = −0.31, *p* = 0.04; 2b) and girls at similar strength (*ρ* = −0.32) but likely failed to reach significance in girls due to the smaller sample size. In contrast to similar relationships between average UH and socialization in boys and girls, opposite trends were observed in average UM; the relationship between UM and socialization scores was positive and insignificant in boys, and it was negative and insignificant in girls (2c). This pattern of results suggests that UM ratio and average UM are not sex-robust indicators of pragmatic ability or social impairment in ASD. UH, on the other hand, appears to be a promising indicator of poor socialization as reported by parents on the VABS, across both sexes.

### Parsing heterogeneity in ASD

Every girl in the ASD group produced UM during at least 40% of filled pauses, and some produced UM during 100% of filled pauses. In contrast, boys with ASD ranged from 0 to 100% of pauses filled by UM vs. UH. This suggests the possibility of multiple subgroups of boys: primarily UMers and primarily UHers. We explored this heterogeneity among boys with ASD by splitting into three equal groups (by UM ratio) and comparing the top and bottom thirds of the boy group to girls. The bottom third of boys produced UH during filled pauses 60% of the time or more, and were categorized as UHers. Boys that produced UM during 78% or more of filled pauses were categorized as UMers. No group differences survived correction for multiple comparisons in a multivariate ANOVA (all *p*s > 0.21; Table 4), indicating that the relationship between filled pause use and Vineland socialization is more dimensional than categorical and may be sensitive to sample size.

**Table 4:** Subgroups of boys with ASD compared to girls with ASD

	Girls	Boy UHers	Boy UMers
Number	16	16	16
Age in years	10.66 (1.55)	10.05 (2.16)	10.04 (2.33)
IQ—full scale	104 (13)	105 (13)	107 (14)
Verbal	106 (15)	106 (13)	111 (15)
Nonverbal	105 (14)	106 (13)	105 (16)
SCQ current	20.81 (4.98)	19.00 (6.64)	18.81 (6.99)
Vineland ABC	79.75 (13.18)	80.06 (12.77)	86.93 (14.44)
Communication	86.38 (12.94)	86.81 (16.10)	90.80 (14.81)
Socialization	73.81 (12.49)	75.13 (14.82)	83.13 (14.53)
ADOS overall	6.31 (2.80)	7.25 (2.05)	5.56 (2.50)
Social affect	6.31 (2.63)	6.81 (2.48)	5.63 (2.42)
RRB	6.50 (3.14)	7.75 (2.11)	6.50 (2.68)

### Specificity of filled pause differences

Finally, we considered the possibility that filled pauses represent just one of many low-level speech differences between boys and girls with ASD. To assess the specificity of variation in filled pause type, we conducted pairwise comparisons across a variety of other linguistic features. Results revealed that girls and boys with ASD did not differ on average total word count, filled pause rate, speech rate, number of turns, duration of turns, response latency, or pitch variation (Table 5).

**Table 5:** Pairwise comparisons between girls and boys with ASD on seven linguistic features (mean and standard deviation (SD))

	Girls	SD	Boys	SD	*z*	Sig.
Total word count	1116.69	533.25	1025.51	472.91	−0.61	*p* = 0.54
Filled pause rate	2.04%	1.5%	2.27%	1.7%	−0.20	*p* = 0.84
Speech rate	0.33	0.04	0.33	0.06	−0.50	*p* = 0.62
Num turns	141.56	45.09	138.08	58.16	−0.43	*p* = 0.67
Duration of turns	436.86	184.99	439.71	195.14	−0.20	*p =* 0.84
Latency to respond	0.83	0.45	0.88	0.33	−0.93	*p =* 0.35
Pitch variation	2.22	1.89	2.12	0.83	−0.81	*p* = 0.42

## Discussion

Subtle linguistic markers influence how parents, teachers, clinicians, and peers perceive an individual’s social skills during everyday conversation. For example, UM is a social pragmatic marker [21] used more often by younger people and women as compared to older people and men, and UH is used relatively more often by older people and men than by younger people and women [59]. Unusually low UM ratios have been reported in children/adolescents with ASD [28], and have been argued to mark difficulties with social communication [24]. In the present study, we asked whether a third variable not considered in prior research—speaker sex—might be important for understanding filled pause differences in children with ASD. Our results showed that girls with ASD and typical girls/boys exhibited higher UM ratios than boys with ASD, while girls in both diagnostic groups suppressed UH relative to their male counterparts. Importantly, filled pause differences in boys and girls with ASD in this study were not attributable to increased social pragmatic ability in girls, as girls and boys in our sample had equivalent social communication skills and comparable autism symptom severity. The present findings suggest that UH suppression and higher UM ratios may serve as “linguistic camouflage” to normalize the way a girl with ASD sounds relative to same-aged typical peers, while elevated rates of UH (relative to UM) may cause boys with ASD to sound particularly atypical. Intentional or not, overtly typical-sounding speech in girls could have some benefits (e.g., allowing a child to more easily “blend in” with classmates), while simultaneously complicating the detection of ASD, leading to missed or delayed diagnosis, and misdiagnoses that are more common in girls than boys [33].

### Sex differences and UM Ratio

This is the first study to show that girls and boys with ASD show sex-specific patterns of filled pause use, and to compare these patterns in girls and boys without ASD. Importantly, girls with ASD in our study did not fill more pauses than boys; they filled them differently. Like typical children and children with specific language impairment [24], girls in the present study used UM during > 70% of all filled pauses. Boys with ASD, in contrast, used UM during only 55% of filled pauses. In contrast to prior research [28], we did not find relationships between ASD symptoms as measured by the SCQ and average UM production in the sample as a whole, nor did we find relationships between average UM and VABS socialization or communication scores. This indicates that even though girls are using typical-sounding speech patterns (as reflected by the UM ratio), it is not necessarily reflected in improved parental perceptions of their social communicative ability.

Why might girls with ASD fill conversational pauses with “typical-sounding” words, and suppress atypical words like UH, despite comparable social communication deficits to boys? One hypothesis is that girls are not using filled pauses as communicative tools, but rather produce higher UM ratios (and lower rates of UH) as a form of unconscious social mimicry or scripting [7, 30, 32, 33, 36]. According to this hypothesis, a girl with ASD might produce typical UM ratios to appear less atypical and improve her chances of successfully integrating during social situations. However, she may not necessarily understand the social meaning behind different filled pause types, and struggle with social communication as much as boys that do not “normalize” their UM ratios. Indeed, our results suggest that girls’ linguistic camouflaging is more successful in some ways than others. Although girls with ASD produced higher UM ratios than boys with ASD due to UH suppression, their overall filled pause rate was still lower than typical participants, resulting in incomplete camouflage. This may be due to the nature of our sample; research-based ascertainment differs from population- or clinic-based approaches, and may have biased our sample toward girls with more pronounced symptoms. Thus, it is possible that girls who have not yet been identified as autistic or referred for an evaluation will engage in more successful (or complete) linguistic mimicry.

Another likely explanation for the sex differences reported here hinges on powerful forces of gender socialization. Indeed, research on typically developing children shows that parent perceptions, play practices, and styles of interaction differ systematically by child sex, with infant girls hearing more language, receiving more eye contact, and having more opportunities to engage in social-emotional interaction than infant boys [9]. These gendered caregiving differences have already been in full effect for 2 to 3 years before most ASD diagnoses are made [6, 9, 15, 17, 23], and may act as pressure on young girls to conform to “girl” expectations that include pragmatic competence. As Goldman [23] pointed out, “children (that are later diagnosed with ASD) are raised like any other children according to their sex. They are perceived as girls or boys and are taught to play, talk, and interact in accordance with the particular gender-based rules of their families.” Thus, perhaps girls with ASD are not using filled pauses communicatively, but instead are responding to forces of gender socialization that expect girls to *sound* less pragmatically impaired, by suppressing UH and producing higher UM ratios. Since girls with ASD and typical girls are subject to the same gender-based influences during the first few years, it follows that their language might be similarly affected. Indeed, we found that typical girls also suppress UH and produce higher UM ratios than typical boys. This is only one possible hypothesis among a confluence of factors that likely relate to the differences observed in this study. Developmental research focused on younger children is needed to chart the causal pathways that lead to this type of linguistic variation in girls.

### Sex differences and UH use

Consistent with prior research documenting UM and UH differences in adult men and women, our results suggest that sex differences in UH production are robust in children; boys in our sample produced more than twice as many UHs (0.84% for boys with ASD, 0.83% for TDC boys) as girls (0.44% for girls with ASD, 0.32% for TDC girls). In addition, we found negative relationships between UH production and perceived social ability across both sexes in ASD; this builds on ﻿a ﻿number of studies linking speech disfluencies to negative social perceptions of speakers [8, 22, 45].

What caused elevated UH in boys with ASD (and typical boys)? Unmeasured variation in basic cognition could explain this finding, since speakers tend to pause more often, and for longer, right before producing syntactically complex utterances or utterances that exceed their linguistic competency [4, 53]. Given that boys and girls in our sample did not differ significantly on IQ, however, basic cognitive differences are unlikely to have caused elevated UH use by boys (IQ was also not a significant predictor of any language variable in our models). Another possible explanation is language-based, since people with uneven language profiles tend to produce more disfluencies [5, 12, 39, 43, 55–57]. Our groups had comparable verbal IQ scores, but it is still possible that boys in our sample had slightly compromised or uneven language abilities relative to girls, who tend to have better language than boys in typical samples as well [44]. Future research is warranted to explore this possibility.

A third explanation, that poor coordination between motor and language systems results in greater UH use by boys with ASD relative to girls, stems from a growing body of research demonstrating subtle motor control differences in ASD [14]. For instance, people who produce high rates of stuttering-like disfluencies often struggle with speech- *and* non-speech motor tasks, suggesting a general deficiency in integrating sensory and motor control information (Smits Bandstraand De Nil, 2009; Webster, 1997; Smith et al., 2012; Louckset al., 2007; Max & Gracco, 2005). Elevated UH rates in boys with ASD may therefore indicate disrupted coordination across motor and language systems, and could provide clues about a global underlying motor-based pathobiology that partially accounts for social communication problems in ASD [14]. However, we know little about how the motor features of ASD may differ by sex; if UH is a marker of asynchrony across motor and language systems that disproportionately affects boys, and elevated UH production drives reduced UM ratios in boys, it could be that motor dysregulation leads to pragmatic problems even when the cognitive aspects of language are grossly intact. We did not measure motor ability in this study, leaving open the possibility that these differences explain some amount of variance, which may or may not vary by sex; future research will address this question.

### Do sex-linked behavior differences necessitate sex-specific diagnostics and treatments for ASD?

Combined with a recent study showing gestural camouflaging in girls with ASD [48], the linguistic camouflage effect identified in this study suggests that subtle sex differences in behavioral domains relevant to ASD (e.g., language) could contribute to girls being missed—or misdiagnosed—due in part to a male-focused conceptualization of ASD and male-normed diagnostic tools. Computational approaches might be particularly useful as an objective way to “see through” various types of social camouflage in the context of screening and diagnosis, and could add to a battery of sex-normed diagnostic and characterization tools for ASD (at least one, the SRS-2, is already normed by sex [13, 31, 33]). In addition to sex-specific identification and characterization tools, behavior-dependent intervention outcome measures may also need to differ by sex. The goal of naturally and automatically integrating information about putative sex differences into the hunt for behavioral biomarkers could lead to creative new approaches that are more sensitive to the way ASD manifests in boys and girls. Notably, our results are consistent with recent large-scale research showing that sex differences in the cognitive and motor profiles of infant siblings of children with ASD are not unique to ASD risk, but rather reflect broader sex differences in the general population [44]. In their conclusion, Messinger and colleagues highlighted the need to compare girls with ASD to typically developing girls (and boys with ASD to typically developing boys). We echo this suggestion.

### Limitations

This study differs from prior research in significant ways. Whereas some studies quantify a variety of disfluencies [42], the current research focused only on UM and UH. Unlike Gorman et al. [24], we counted multiple contiguous instances of UM and UH (i.e., if UM was repeated more than once during a filled pause, we counted it more than once. This allowed us to capture stuttering-like filled pauses). Our sample included the largest number of girls with ASD to date, reflecting a 4:1 ratio of boys-to-girls (close to the generally accepted average ratio in ASD). However, we did not include equal numbers of girls and boys with ASD, which led to power issues when examining variables within each sex separately, and we included only a small sample of typically developing control participants. As with some prior studies, we only focused on the interview section of the ADOS-2, and only used Module 3 recordings—future research should expand to new age ranges, ability levels, and ADOS-2 sections and modules.

An inherent limitation of analyzing language produced during semi-structured evaluations is that clinicians are free to ask questions in any order, or to skip questions completely if appropriate from a clinical perspective. Although all clinicians in the current study were research-reliable PhD-level psychologists administering the ADOS in a single center, it is likely that not all children were given probes in the same order. In addition, all clinicians in this study were female, which means that language samples from male participants may have included more UM than if they had been interviewed by a male clinician [38]. Future research will systematically control for the sex of the interlocutor, to assess effects of sex match vs. mismatch on language variables in individuals with ASD. Finally, it is possible that clinicians differed in how often they asked questions vs. used open-ended comments to start conversations. These variations were limited to the extent possible, but are inherent in any research that relies on ADOS evaluations.

We did not examine the influence of co-occurring disorders or dimensions, such as anxiety, attention deficit/hyperactivity disorder, and executive dysfunction in ASD, on filled pause use. These nuances are ripe avenues for future research. Consistent with findings from other studies of children with ASD, our analyses revealed no relationships between age, IQ, and filled pause type. The lack of an age effect may be due to the age range of our sample (which does not extend into adulthood). Future research with older men and women with ASD is needed to elucidate how our findings change across the lifespan.

The present research represents a number of advances over prior studies. Our results are based on longer language samples than prior research (e.g., 20 min vs. 60 s in [28]). We analyzed UM/UH data from the largest group of individuals with ASD reported to date (65 in this study vs. 50 in [24] and 24 in [28]). Importantly, this is the very first study to examine sex differences in speech disfluencies in children with ASD, made possible by the inclusion of a relatively large number of girls with ASD (25% of our sample vs. 10% and 12.5% in prior work). This is also the first time UM and UH have been explored in typically developing boys and girls, who were found to produce even higher UM ratios than adult men and women [58–60]. Finally, the present research shows that the conclusions of past studies of UM and UH in children with ASD were not entirely wrong*.* Rather, they were correct for boys with ASD, and incorrect for girls.

### Future directions

Prior research shows that children and adolescents with ASD produce more fillers when cognitive demands are high [28], which for individuals with social challenges, may occur more often when discussing social topics. Although others have shown that UM is used relatively less frequently by children with ASD across a variety of tasks [26], future research should specifically compare children's responses to highly social questions and less-social questions (e.g., about objects or interests). The discrepancy in UM and UH production between different question types that vary by social load may be even more informative than relative usage collapsed across an entire interaction. Due to constraints related to the semi-structured conversation format employed in the ADOS-2, we plan to conduct this subsequent experiment in a more controlled format.

Interestingly, Irvine and colleagues suggested that reduced UM use in ASD might contribute to the perception of speech as pedantic or stilted [28]. Based on our current findings, we propose that elevated UH during conversation might in fact drive the impression of pedantry, more so than reduced UM. In fact, high rates of UH (without corresponding increases in UM) might be so unusual in young people [59] that they contribute to some individuals with ASD sounding older than they appear (thus, the “little professor”). Future studies with older and larger samples of individuals with ASD, as well as human raters, are needed to test this hypothesis.

We found that filled pauses vary by sex in ASD, but the everyday consequences of these variations are unknown. Do typical-gendered language patterns in girls with ASD contribute to older age at diagnosis, missed diagnoses, or misdiagnosis? Given that early detection and early intervention are critical for maximizing functional outcomes in ASD [29], issues of under-referral and inadequate measurement for girls with ASD are not trivial. Outside of diagnostic issues, it is possible that suppressed UH and typical UM ratios in girls correlate with the perception of social normalcy by peers, thus helping to establish or maintain friendships. In addition, perhaps girls with ASD suppress UH more when talking to peers than when talking with adults, mimicking typical behavior and increasing their chances of social affiliation in classrooms or on playgrounds. Future studies in naturalistic settings, with a variety of interlocutors, are needed to answer these questions.

## Conclusion

ASD experts make diagnostic decisions based on observable behavior, and subtle differences in how a child moves or talks will influence the way they are perceived. Gender socialization or social mimicry may lead to “camouflaged” behavior in girls with ASD, which, combined with widely held gender biases about how girls and boys *should behave* and true biological sex differences, likely complicate efforts to effectively identify and treat boys and girls with ASD. Recent attempts to reduce bias by directly sampling behavior and using objective, computational measurement tools hold promise over existing parent report and clinician rating scales [48], but even these new tools will likely be influenced by variables such as age, sex, gender socialization, socio-economic status, physical and mental health, and home and cultural environment. The findings reported here, identifying “linguistic camouflage” in girls with ASD, highlight the importance of continued commitment to understanding the complex web of biological and environmental factors that influence ASD emergence and presentation.

## Abbreviations

ADOS-2: Autism Diagnostic Observation Schedule-2nd Edition
ASD: Autism spectrum disorder
DAS: Differential Abilities Scales-II–2nd Edition
SCQ: Social Communication Questionnaire
TDC: Typically developing control
VABS: Vineland Adaptive Behavior Scales
